# Provocative mesenteric angiography for occult gastrointestinal bleeding: a systematic review

**DOI:** 10.1186/s42155-023-00386-7

**Published:** 2023-08-17

**Authors:** Siddhi Hegde, Patrick D. Sutphin, Omar Zurkiya, Sanjeeva P. Kalva

**Affiliations:** Division of Interventional Radiology, Department of Radiology, Massachusetts General Hospital, Harvard Medical School, Boston, MA USA

**Keywords:** Provocative angiography, Heparin, Thrombolytics, Occult gastrointestinal bleeding

## Abstract

Occult gastrointestinal bleeding (GIB) is a challenge for physicians to diagnose and treat. A systematic literature search of the PubMed and Embase databases was conducted up to January 1, 2023. Eligible studies included primary research studies with patients undergoing provocative mesenteric angiography (PMA) for diagnosis or localization of occult GIB. Twenty-seven articles (230 patients) were included in the review. Most patients (64.8%) presented with lower GIB. The average positivity rate for provocative angiography was 48.7% (58% with heparin and 46.7% in thrombolytics). Embolization was performed in 46.4% of patients, and surgical management was performed in 37.5%. Complications were rare. PMA can be an important diagnostic and treatment tool but studies with high-level evidence and standardized protocols are needed to establish its safety and optimal use.

**Supplementary Information:**

The online version contains supplementary material available at 10.1186/s42155-023-00386-7.

## Background

Occult gastrointestinal bleeding (GIB), defined as bleeding of unknown origin that persists or recurs after an initial negative diagnostic evaluation, presents a diagnostic challenge for physicians. Occult GIB in hemodynamically unstable patients is a common cause of referral to interventional radiology (IR). Mesenteric angiography is usually performed following confirmation of bleeding on CT angiography or nuclear scintigraphy. It has a diagnostic yield of 40%-86% but requires a minimum bleeding rate of 0.5–1.0 mL/min [1]. Hemodynamic instability and significant blood product transfusions are predictors for positive angiography. Packed red blood cell (PRBC) transfusion increases the likelihood of positive angiography by 30% per unit within a 24-h period [2].

A positive initial diagnostic test may be followed by a negative angiography. Several causes are hypothesized- intermittent bleeding (vasospasm), slow rate of bleed, and cessation of bleed. Provocative angiography, a technique that involves injecting a provocative drug into the suspected bleeding site to precipitate active bleeding, has been proposed to identify the source of occult GIB. Various pharmacological drugs such as heparin, vasodilators, thrombolytics and recently, vasoconstrictors have been hypothesized to have efficacy in inducing, prolonging, or augmenting active bleeding. The safety and efficacy of this technique remains a matter of debate. This systematic review aims to evaluate the technique and clinical outcomes associated with provocative mesenteric angiography (PMA) for occult GIB.

## Methods

Two authors (SH, SK) performed independent systematic reviews according to the PRISMA statement. The Critical Appraisal Skills Program (CASP) for cohort studies was used to assess the quality of the selected studies. Three items (results of the study, how precise they are and what are the implications of the study for practice) of the CASP tool were left out of the critical appraisal. A follow-up of at least one month was considered appropriate to judge the effect on diagnosis and cessation of bleed. The Oxford Centre for Evidence-Based Medicine Levels of Evidence (OLOE) were determined for all studies, where level 1 is the highest level of evidence and level 5 is the lowest.

### Search strategy

A systematic literature search, up to 1 January 2023, of the PubMed and Embase databases was undertaken with the aid of a clinical librarian, using medical subject headings and free-text words concerning PMA for occult GIB (Supplementary information). No language or time period restrictions were applied. Retrieved titles and abstracts were screened for relevance by two authors (SH and SK), and selected studies, case reports, and cohort studies were fully assessed to fulfil eligibility criteria. Articles were checked for overlapping data, and, when identified, the smaller study was excluded. Reference lists of all included articles were screened for additional eligible articles.

Studies were excluded based on the listed criteria. The authors (SH and SK) independently assessed each study to determine whether it met the pre‐defined selection criteria. Disagreements during the search and selection process were resolved by discussion, and, if needed, a third reviewer was consulted to reach a consensus.

### Eligibility criteria

#### Inclusion criteria

- Primary study design with patients undergoing provocative angiography with any drug for the purpose of diagnosis of occult GIB or its localization.

#### Exclusion criteria

- Studies involving patients aged < 18 years.
- Article text in non-English
- Review articles

### Data collection and definitions

Two authors (SH, SK) extracted data using standardized forms. The following baseline data were collected: author and institution, publication date, journal, study design, number of patients, age and sex, number of prior negative angiograms, and length of follow-up.

Information on the indication for provocative angiography, procedural details (doses, provocative drug(s), and sequential use of heparin/vasodilators/thrombolytics, artery where the drug was injected), and type of embolization material were recorded. The following outcome data were collected: reported positive rates following provocative angiography (success rate), the number of patients receiving embolization and surgery, diagnosis, bleeding vessel, complications after provocative angiography, and rebleeding in negative provocative procedures. The Society of Interventional Radiology (SIR) Classification System for Complications by Outcome was used [3].

### Statistical analysis

Outcomes are displayed as reported in the original article. No meta-analysis was performed because of substantial heterogeneity between studies. No statistical analysis was done as outcomes were reported using varying outcome measures and techniques. Data were tabulated as numbers of patients and their weighted average or mean, or sum values.

## Results

**Fig. 1:**
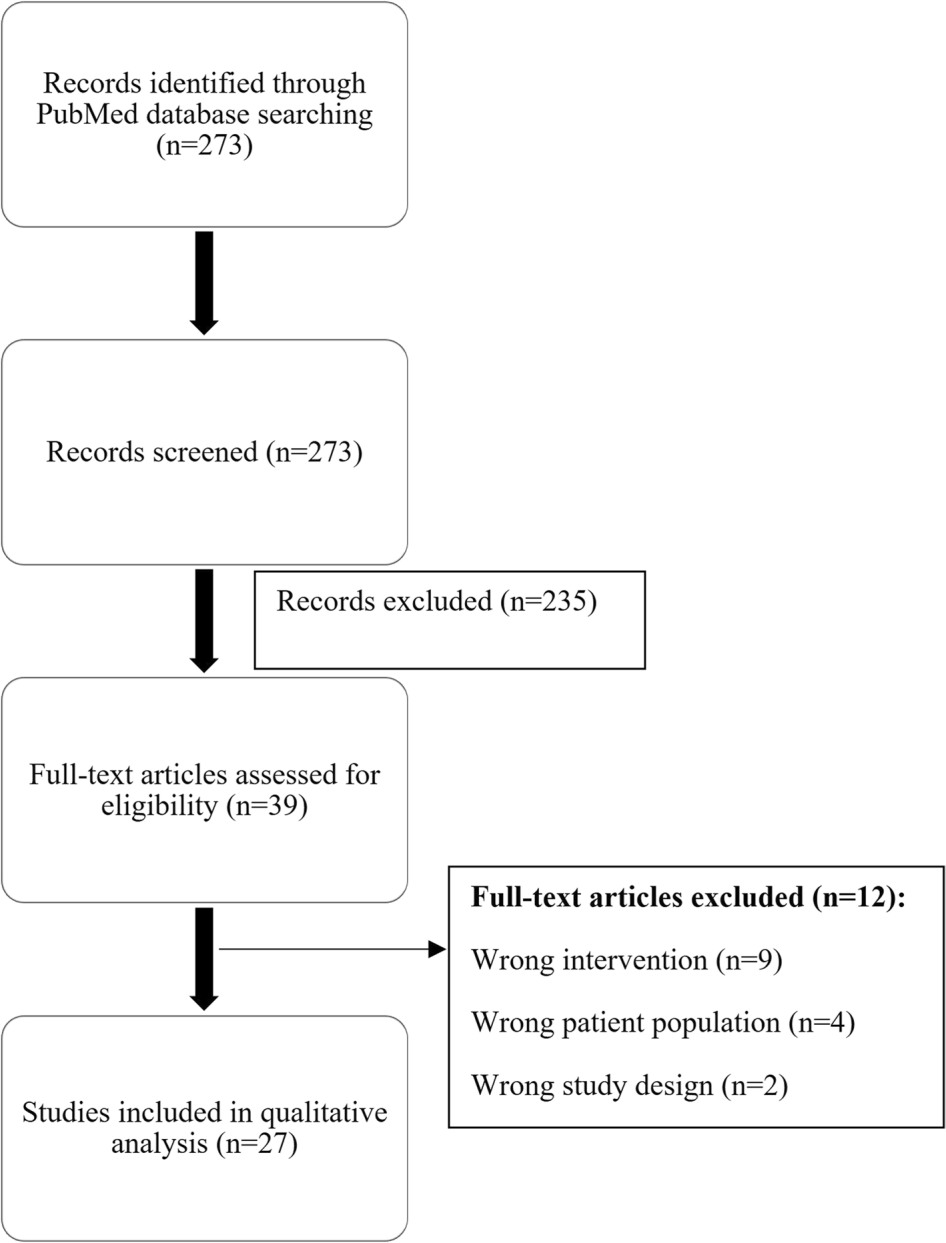
PRISMA flowchart of selection of articles for review

A total of 273 articles were identified. After screening by title and abstract, a further 235 articles were excluded. After a full-text assessment of the remaining 38 articles, 27 studies (15 retrospective database review studies, one prospective cohort, 10 case reports, and 1 case series) met the eligibility criteria and were included in the review (Fig. 1).

### Quality assessment

Table S1 (Supplementary information) summarizes the quality of each cohort study. The 16 cohort studies were considered to provide level 2b evidence. All 11 case reports (including 1 case series) were considered level 4 evidence.

### Baseline characteristics

The twenty-seven articles included a total of 230 patients, of whom 149 (64.8%) patients presented with lower GIB (complaints of melena, hematochezia), one patient (0.43%) with upper GIB, 26 (11.3%) patients with both upper GIB and lower GIB and 54 (23.5%) patients with GIB from unknown/unreported region. Duration of these complaints ranged from 2 weeks up to 4 years, with some patients (58/230, 25.2%) presenting with recurrent episodes of GIB. 57.1% (4/7) studies reported the duration between these recurrent episodes (mean duration between episodes: 13.6 months).

### Provocative angiography with heparin only

The four retrospective cohorts (database review) and two case reports included 29 and 2 patients, respectively [4–9].

#### Patients—presentation, prior negative studies

**Fig. 2:**
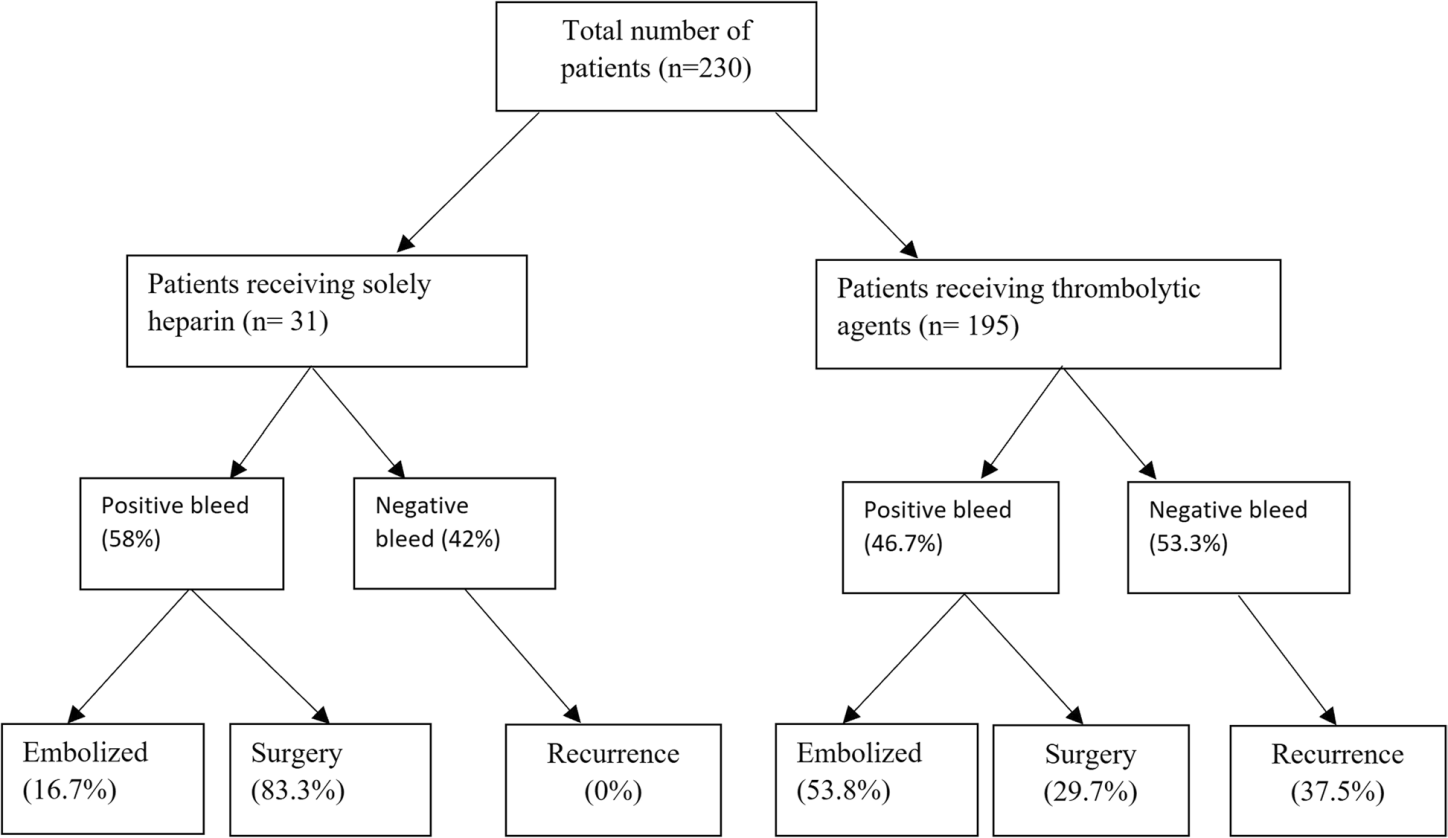
Flowchart of included patients

The six articles (Table 1) included a total of 31 (13.4% all patients) patients (Fig. 2), of whom 27 (87.1%) patients presented with lower GIB (complaints of melena, hematochezia), 1 patient (3.2%) with both upper and lower GIB, and 3 (9.7%) patients with GIB from unknown/unreported region. Duration of these complaints ranged from 6 months up to 4 years with some patients presenting with recurrent episodes of GIB.

**Table 1:** Studies that used only Heparin for provocative angiography

Study (Year)	No. of patients	Heparin/ Vasodilator use (systemic/intravenous)	Detected bleed (Positive provocative bleed)	Embolized	Surgery	Complications related to provocative therapy (yes/no)	Recurrent bleed in negative provocation patients	Follow-up (months)	Reason for PMA	Prior negative interventions
Brünnler (2008) [4]	13	Heparin	9 (69%)	-	Yes	No	-	-	Recurrent GIB of obscure origin	Scintigraphy with 99mtc-marked red blood cells as a diagnostic procedure
Drezdzon (2022) [5]	1	Heparin/ Vasodilators	0 (0%)	No	Yes	No	No	-	LGIB	Diagnostic colonoscopy, nasogastric lavage and subsequent EGD
Mernagh (2001) [6]	12	Heparin, Papaverine	6 (50%)	No	Yes (100%)	No	No	-	Obscure chronic GIB	Conventional angiography 1x
Nozawa (2022) [7]	2	Heparin	2 (100%)	Yes (100%)	No	No	No	-	Acute LGIB	Conventional angiography 1x
Lee (2012) [8]	2	Heparin	1 (50%)	-	-	Yes (*n* = 1)	-	0–5	Acute UGIB or LGIB	Conventional angiography 1x
Hasaj (2004) [9]	1	Heparin	0 (0%)	Yes	No	No	No	17	Recurrent UGIB and LGIB	3 × angiographies with or without heparin provocation
**Total**	**31**	**Heparin (100%)**	**18 (58%)**	**3 (16.7%)**	**15 (83.3%)**	**1 (5.5%)**	**No (100%)**	7.3*		

*Calculated weighted average

Patients mostly had single negative angiography (14/31, 45.2%) before provocation. One patient (3.2%) underwent multiple negative angiography studies; mean: 1.5 angiograms per patient, range: 1–3 angiograms [9]. One author also reported investigating one patient (*n* = 1/31, 3.2%) with colonoscopy and esophagogastroduodenoscopy (EGD), and another author reported using tagged red-blood-cell (RBC) scintigraphy (*n* = 13/31, 41.9% patients) [4, 5].

#### Protocol

One study (16.7%) provided detailed stepwise protocol mentioning the technique and materials required for angiography [6]. Low-dose heparin (less than 5000 IU) was used for intra-arterial injection (*n* = 2 studies, mean = 3000 IU) [6, 7]. Papaverine was given intra-arterially (65 mg) as a vasodilator, followed by heparinization if the angiogram was negative [5, 6].

#### Positive rate

Heparin, as the sole agent, demonstrated 58% positivity in provoking GI bleeds (Table 1). With a median follow-up time of 7.3 months, no recurrent bleeding was reported in patients with either a positive or a negative study following provocation; four studies did not provide information on their follow-up period.

#### Location of bleed

The source of bleeding was reported in four studies (4/6, 66.7%). Extravasation was seen from the small intestine (*n* = 6), transverse colon (*n* = 1), mid-ascending colon (*n* = 1), cecum (*n* = 1).

#### Diagnosis

Three studies (50%) reported underlying disease responsible for bleeding. Mernagh et al. diagnosed angiodysplasia, vascular malformations and active Crohn's disease [6]. Hasaj et al. diagnosed hemosuccus pancreaticus (hemorrhage through the pancreatic duct into the duodenum), and Drezdzon et al. diagnosed multiple non-dysplastic adenomas [5, 9].

#### Treatment

Three patients (16.7%) underwent embolization. The type of embolic material used was reported in only one study, and included gelatin sponge, polyvinyl alcohol particles, metallic microcoils and n-butyl cyanoacrylate (nBCA). Fifteen patients (*n* = 15, 83.3%) underwent surgical management (Table 1). One study reported the surgery performed as hand-assisted segmental transverse colectomy [5].

#### Complications

Complications were reported in one study (1/31, 3.2%) (Table 1) [8]. The patient had a major category D complication i.e., a massive intestinal hemorrhage requiring transfusion. There were no deaths reported.

### Provocative angiography with thrombolytic drugs

The eleven cohort studies and nine case reports (including one case series) included 195 patients (84.8% all patients), of whom 145 underwent provocation with tissue plasminogen activator (tPA) (72.8%), 37 (18.6%) with Urokinase and 13 (2.6%) with Streptokinase [2, 10–28].

#### Patients—presentation, prior negative studies

The twenty articles included a total of 195 patients (Table 2), of whom 137 (70.2%) patients presented with lower GIB (complaints of melena, hematochezia), 1 patient (0.5%) with upper GIB, 23 (11.8%) patients with complaints of both upper GIB and lower GIB and 34 (17.4%) patients with GIB from unknown/unreported region. Duration of these complaints ranged from 2 weeks to 4 years with some patients presenting with recurrent episodes of GIB.

**Table 2:** Thrombolytic/other agents' use

Study (Year)	No. of patients	Heparin/ Vasodilator use (systemic/catheter-delivered)	Thrombolytic use (intra-arterial)	Detected bleed (Positive provocative bleed)	Embolized	Surgery	Complications related to provocative therapy (yes/no)	Recurrent bleed in negative provocation patients	Follow-up (months)	Reason for PMA	Prior negative interventions
1. Johnston (2007) [25]	1	Heparin, (Papaverine- intraarterial)	tPA	1 (100%)	Yes (100%)	No	No	No	10	Chronic LGIB	Conventional angiography 1x
2. Remzi (2003) [24]	1	Heparin	tPA	1 (100%)	No	Yes	No	No	18	Recurrent LGIB	Conventional angiography 2x
3. Ryan (2001) [13]	17	Heparin, (Tolazoline-intra-arterial)	tPA	6 (37.5%)- 8 (50%)	Yes (3/8)	Yes (1/8)	No	Yes (5/8)	3–34	Occult LGIB	Red blood cell scintigraphic studies and conventional angiography (*n* = NR)
4. Shetzline (2000) [23]	1	Priscoline, (Heparin -intravenous)	tPA	1 (100%)	Yes	No	No	No	8	Recurrent GIB of obscure origin	Colonoscopy, EGD, upper endoscopy with small bowel enteroscopy, 99mTc scintigraphy
5. Kokoroskos (2020) [16]	23	Heparin, Nitroglycerine	tPA	7 (30%)	Yes (*n* = 4)	Yes (*n* = 3)	No	Yes (*n* = 2/16)	-	GIB (upper and lower)	Conventional angiography 1x
6. Meade (2020) [27]	1	Heparin (systemic and also given intravenous)	tPA	1 (100%)	Yes	No	No	No	15	Obscure UGIB	12 OGDs, 3 colonoscopies, 3 VCEs, 2 enteroscopies, 1 duodenoscopy, 1 endoscopic ultrasound, 2 CT angiograms, MR enterography, and MR cholangiopancreatography
7. Thiry (2022) [18]	36	Nitroglycerin, Heparin (unspecificed)	tPA	16 (44%)	Yes (100%)	No	No	Yes (*n* = 12/20, 60%)	1	LGIB	Conventional angiography
8. Wu (2013) [26]	1	-	tPA	1 (100%)	Yes	No	No	No	-	LGIB	Serial angiograms (*n* = NR)
9. Zurkiya (2015) [15]	19	Heparin, Verapamil/Nitroglycerin	tPA	5 (26%)	-	Yes	-	-	-	Recurrent GIB of obscure origin	Normal or non-localizing endoscopic and imaging findings, including angiographic findings
10. Kim (2010) [2]	36	Heparin, (Tolazoline/Papaverine-intra-arterial)	tPA (Alteplase)	11 (31%)- 12 (33%)	Yes (*n* = 10/11, 92%)	Yes (*n* = 1/10)	No	Yes (*n* = 11/25, 44%)	12	Recurrent LGIB	Mesenteric angiogram (*n* = 24/29, mean 1.2 angiograms per patient; range: 1–4)
11. Widlus (2007) [14]	9	-	tPA (Reteplase)	8 (89%)	Yes (*n* = 5)	Yes (*n* = 1)	No	No	3–44	Recurrent, massive, LGIB of obscure origin	3 vessel diagnostic arteriography (1x)
**Total/Averages**	**145**	**Heparin (9/11)** **Nitroglycerin (3/11)**	**tPA (*n* = 11, 100%)**	**59 (40.7%)**	**37 (62.7%)**	**12 (20.3%)**	**No (100%)**	**30 (34.9%)**	**9.5**^**a**^		
12. Koval (1987) [10]	10	Heparin, Tolazoline	Streptokinase	8 (80%)	Unclear	Yes (*n* = 5)	Yes	Unclear	36	LGIB	99tc-labeled red blood scans
13. Rösch (1982) [28]	3	Heparin, Tolazoline	Streptokinase	3 (100%)	No	Yes (100%)	Yes	Yes (*n* = 1)	8–12	Recurrent LGIB	1) angiography 1x 2) sigmoidoscopy, colonoscopy 3) angiography 6x
**Total/Averages**	**13**	**Heparin (2/2)** **Tolazoline (2/2)**	**Streptokinase (*n* = 2, 100%)**	**11 (84.6%)**	**3 (27.2%)**	**8 (72.7%)**	**Yes (100%)**	**Yes (33.3%)**	**30**^**a**^		
14. Malden (1998) [19]	10	Heparin	Urokinase	4 (40%) studies within 4 h, 3 (30%) at 8–24 h after initiation	Yes (*n* = 1)	Yes (*n* = 2)	No	Yes (*n* = 5/10)	5–35	LGIB	Esophagogastroduodenoscopy, colonoscopy, small-bowel examination, and conventional angiography (*n* = NR)
15. Miller (1999) [22]	1	-	Urokinase	0 (0%)	No	Yes	No	No	-	Obscure GIB	Upper endoscopy, colonoscopy, and small bowel enteroscopy, upper and lower GI and small bowel barium studies, enteroclysis, radionuclide bleeding studies
16. Bloomfeld (2000) [12]	7	Heparin, Tolazoline	Urokinase	2 (29%)	No	Yes (2/2)	Yes (*n* = NR)	Yes (*n* = 1)	7–34	Recurrent GIB of obscure origin	Angiography 1x
17. Cohn (1998) [11]	5	Heparin (*n* = 2) and Tolazoline hydrochloride (*n* = 1)	Urokinase (*n* = 1)	4 (80%)	-	Yes (*n* = 1)	Yes (*n* = 1)	-	-	LGIB	Conventional angiography 1x
18. George (1991) [21]	1	Heparin, Vasodilator	Urokinase	0/3 (0%)	No	Not possible	No	-	-	Recurrent massive GIB of obscure origin	Upper and lower endoscopy, intravenous sulfur colloid and tagged red cell bleeding scans
19. Glickerman (1988) [20]	1	Heparin, Tolazoline	Urokinase	1 (100%)	No	Yes	Yes	No	9	Recurrent LGIB	Angiography 4x
20. Kariya (2020) [17]	12	Heparin(*n* = 2), Nicardipine(*n* = 3)/ Alprostadil(*n* = 1)/ Isosorbide(*n* = 1)	Urokinase	6 (50%)	Yes (*n* = 6)	Yes (*n* = 1)	No	Yes (*n* = 2)	-	LGIB	Colonoscopy, conventional angiography (*n* = NR)
**Total/Averages**	**37**	**Heparin (6/7)** **Tolazoline (3/7)**	**Urokinase (*n* = 6, 100%)**	**20 (54.1%)**	**7 (35%)**	**7 (35%)**	**No (85%)**	**8 (47%)**	**9.5**^**a**^		
**Totals**	**195**	**Heparin (17/20)** **Tolazoline (7/20)** **Nitroglycerin (3/20)**	**tPA (145, 64.2%)** **Streptokinase (13, 6.7%)** **Urokinase (37, 19%)**	**91 (46.7%)**	**49 (53.8%)**	**27 (29.7%)**	**8 (3.5%)**	**39/104 (37.5%)**	**10.9**^**a**^		

^**a**^Calculated weighted averages

Patients mostly had single negative angiography (137/195, 70.3%) before provocation. Some patients (26/195, 13.3%) underwent multiple negative angiography studies (mean 1.6 angiograms per patient; range: 1–6). Several authors (9/21, 42.8%) also report concurrently investigating patients (73/195, 37.4%) with endoscopy, colonoscopy, enteroscopy, oral contrast studies, or tagged RBC scintigraphy (5/9, 55.5%).

#### Protocol

Eleven studies (52.4%) provided detailed stepwise protocols mentioning the technique and materials required for angiography (Table S 2). Mean tPA dose was 24 mg. The mean urokinase dose was 592,500 IU (4 studies). A dose of 60,000 IU of streptokinase was given in 1 study. The upper limits of the range of administered doses (maximum dose) were taken to be the overall dose for the study.

Several authors (17/20, 85%) concurrently used intra-venous/systemic heparinization in 171 (171/195, 87.7%) patients. Among the low-dose (< 5000 IU) heparinized patients, 5000 IU was the most common dose (range: 500-5000 IU; *n* = 140/171, 81.8%). Among the high-dose (> 5000 IU) heparinized patients (*n* = 5 studies; 31/171 patients, 18.1%), 10,000 IU was the only dose administered. The upper limits of the range of administered doses (maximum dose) were taken to be the overall dose for the study.

Other vasodilators such as papaverine, tolazoline, nitroglycerin, verapamil, nicardipine, alprostadil and isosorbide were mentioned being used. Heparin was the most used drug (85%), followed by tolazoline (7/20 studies, 35%) and nitroglycerin (3/20, 15%).

#### Positive rate

Despite being the most widely used agent, tPA demonstrated the lowest positivity rate (40.7%) (Table 2). Streptokinase (84.6%) and urokinase (54.1%) demonstrated comparable positivity rates, despite the limited number of studies (*n* = 2 and *n* = 6, respectively). The overall positivity rate for thrombolytics was 46.7% (91/195). Of the negative provocation patients (*n* = 104), 37.5% (*n* = 39) reported having a recurrent episode of bleeding within the follow-up period. Most of the negative provocation patients who were treated with urokinase suffered a recurrent bleed (47%).

With a median follow-up time of 10 months, recurrent bleed was reported in 5 provoked bleed patients (within 30 days from the provocative procedure), although 6 studies did not provide information on their follow-up period.

#### Location of bleed

The source of bleeding was reported in 11 studies. It was determined to be the superior mesenteric artery (SMA) (or its branch) in most of the patients (*n* = 9). The other reported sources were the inferior mesenteric artery (IMA), common hepatic artery (CHA), gastroduodenal artery (GDA), right and left colic arteries, splenic artery, ileocolic and pancreaticoduodenal collaterals. Extravasation was seen from the small intestine (*n* = 10), ascending colon (*n* = 1), descending colon (*n* = 3), cecum (*n* = 2), sigmoid colon (*n* = 4) and the large bowel (*n* = 19).

#### Diagnosis

Seven authors (33.3%) reported the underlying disease responsible for bleeding. They found angiodysplasia, chronic ulcers, diverticulosis, portal hypertension and malignancy (carcinoid tumor) to be the main causes [2, 10, 12, 20, 21, 24, 28].

#### Treatment

53.8% (49/91) patients underwent embolization. The type of embolic agent used was reported in 9 studies (9/21, 42.8%) and comprised mostly of metallic microcoils followed by gelatin sponge, polyvinyl alcohol particles, and n-butyl cyanoacrylate (nBCA). The types of surgeries were mentioned in 4 studies (4/21, 19%). 27 patients underwent surgical management (2 hemicolectomies and 2 resections of bowel segments and 1 laparotomy).

#### Complications

Complications were reported in 5 studies (*n* = 8/195 patients, 4.1%) and consisted mainly of bleeding at the access site (*n* = 4 patients) and hemorrhage requiring transfusion (*n* = 2 patients). Major category D complications occurred in 3 patients (3/8, 37.5%) and a category C complication in 1 patient (1/8, 12.5%). Minor category B complications occurred in 1 patient (1/8, 12.5%) and category A complications in 3 patients (3/8, 37.5%). One category D complication (1/8, 12.5%) i.e., ischemia and/or perforation was attributed to embolization post-provocative angiography with thrombolytic drugs [2]. There were no deaths reported.

There were no complications associated with tPA use (0/11 studies, 0%). All studies that involved the use of streptokinase (2/2, 100%) had associated massive hemorrhage (2/9 patients, 22.2%). 3 (3/6, 50%) studies that used urokinase reported complications, all of which were prolonged access site bleeding. All 3 were in combination with heparin and the heparin infusion was incriminated as the responsible agent in 2 such studies [12, 28].

### Provocative angiography with norepinephrine

One study used norepinephrine (NE) on four patients (4/230, 1.7%) as the provocative drug [29]. Patients presented with symptoms of lower GIB (4/4, 100%). Patients underwent a single negative angiography and at least three unsuccessful endoscopic therapies prior to the provocative angiography. The maximum dose of NE administered was 40 µg (mean = 27.5 µg). The bleeding was localized in 75% patients (*n* = 3/4) and determined to be the right colic artery (*n* = 1), ileocolic artery (*n* = 1) and IMA (*n* = 1). One patient was diagnosed to have inflammatory bowel disease (1/4, 25%). In all cases, embolization with microcoils was performed (3/3, 100%). Super-selective embolization failed in one patient, leading to a category D complication (1/3, 33.3%) i.e., mesenteric ischemia necessitating hemicolectomy.

### Overall results

The 27 articles included a total of 230 patients, 149 (64.8%) presented with lower GIB, 1 (0.43%) with upper GIB, 24 (10.4%) with complaints of both upper GIB and lower GIB and 56 (24.3%) with GIB from unknown/unreported region. Duration of these complaints ranged from 2 weeks to 4 years with some patients presenting with recurrent episodes of GIB.

On an average across all types of drugs, provocative angiography had a 48.7% (112/230) positivity rate. Of the negative provocation patients (*n* = 118), 33.1% reported having a recurrent episode of bleeding within the follow-up period. Most of the provoked bleeds were from the SMA or one of its branches. 10 authors reported the underlying disease responsible for bleeding.

In total (cohort studies and case reports), 52 patients underwent embolization (46.4%), and 42 patients (37.5%) underwent surgical management after diagnosis/localization of the bleed with PMA.

Heparin was responsible for 33.3% (3/9) of all complications. tPA was the safest drug with 0% complications being reported, followed by urokinase. In contrast, streptokinase was highly unsafe with category D complications in both studies that used it. Overall complications were reported in 7 studies (*n* = 10 patients) and consisted mainly of bleeding at the access site (*n* = 4) and hemorrhage requiring transfusion (*n* = 5). Major category D complications occurred in 5 patients (5/10, 50%) and a category C complication in 1 patient (1/10, 10%). Minor category B complications occurred in 1 patient (10%) and category A complications in 3 patients (3/10, 30%). Two category D complications (2/10, 20%) i.e., ischemia and/or perforation were attributed to embolization post-provocative angiography. There were no deaths reported.

## Discussion

The use of provocative angiography for GIB was first reported in 1982 and has gained significant attention in recent years with most studies conducted since 2010 (42.3%) [28]. This systematic review evaluated the clinical outcomes and safety of PMA for occult GIB. The review found that PMA had a mean positivity rate of 48.7%, and embolization of the bleeding vessel(s) was performed during the same procedure in 46.4% of patients, highlighting its therapeutic benefits. However, patients with negative results had a 33.1% incidence of recurrent bleeding within the follow-up period, indicating that PMA is not always successful in identifying the source of GIB.

**Fig. 3:**
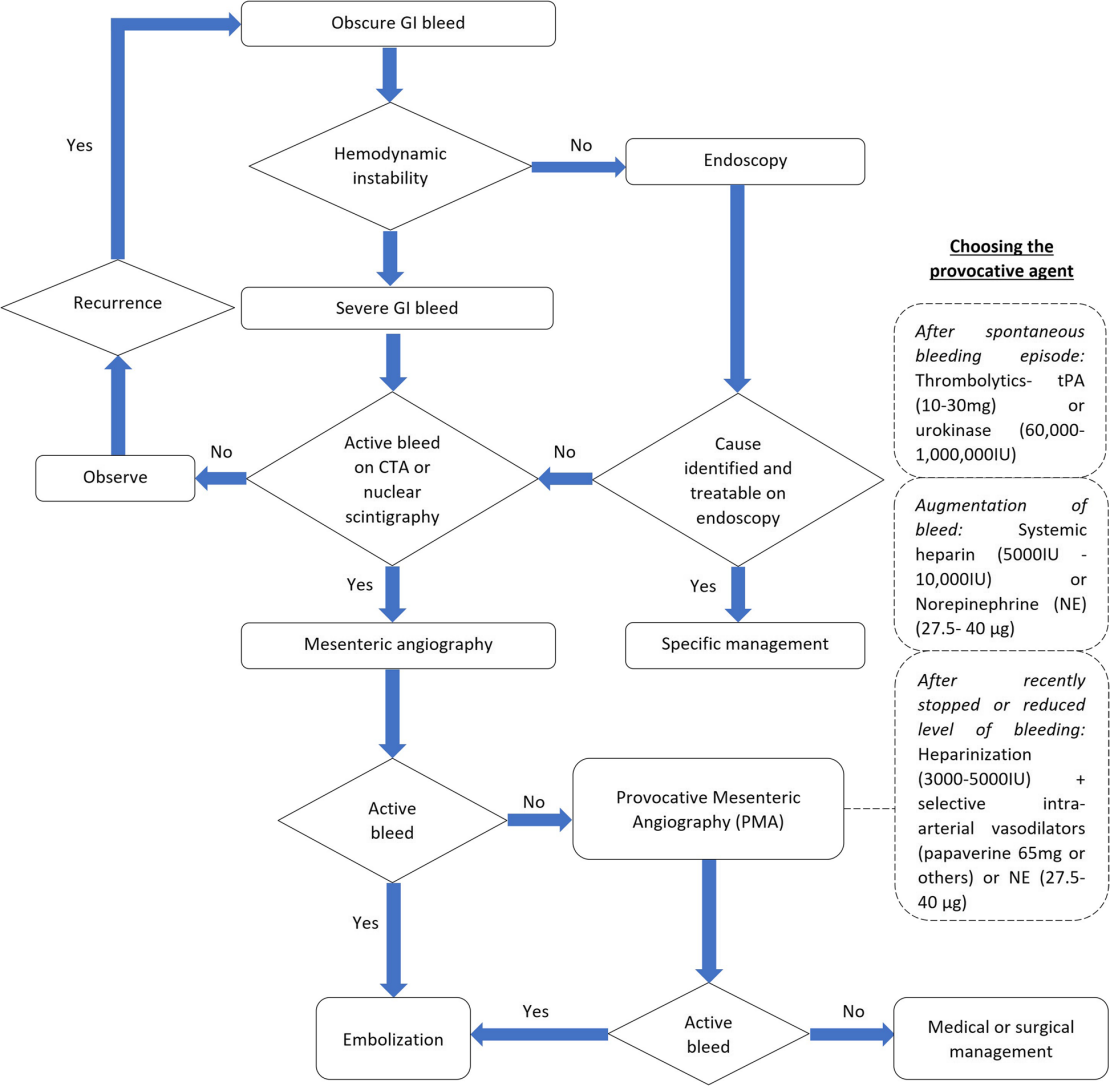
Algorithm for management of acute gastrointestinal bleed

The review also found that the types of pharmacological provocation used in PMA have been variable, with differences in patient presentation, physician experience, and evolving availability of thrombolytic therapy (Fig. 3). The timing of the provocation may help choose the right pharmacologic agent. Heparin, as an anticoagulant, prevents the formation of thrombus during systemic administration and may augment active bleeding on angiography. Fibrinolytic drugs activate plasminogen to plasmin, which degrades the formed thrombus, thus leading to bleeding from a recently thrombosed vessel. Vasodilators cause the smooth muscles in the blood vessel walls to relax [28]. Norepinephrine is a vasoconstrictor which, through alpha-1-induced global vasoconstriction and resulting increase in blood pressure, can provoke contrast media extravasation on angiographical administration [29]. Provocation early after a spontaneous bleed is theoretically more likely to induce bleeding because the thrombus should be less organized and more susceptible to thrombolysis [12]. Heparinization in combination with selective intra-arterial vasodilators may facilitate an angiographic diagnosis in recently stopped or low-level bleeding. Vasodilators alone may be effective during the early vasospastic phase [28].

Angiography is better suited than endoscopy or surgery to achieve initial hemostasis in severe cases [30]. In such cases, embolization of the bleeding vessel(s) can be performed during the same procedure, providing immediate and effective treatment. Embolization of the bleeding vessel(s) was performed during the same procedure in 46.4% of patients, highlighting the therapeutic benefits of this technique. Patients with negative results had a 33.1% incidence of recurrent bleeding within the follow-up period, indicating that PMA is not always successful in identifying the source of GIB.

The overall data suggest that provocative angiography can be performed safely. Complications associated with the procedure were generally rare, but prolonged bleeding (> 30 min) at the access site was the most common complication. Post-embolization ischemia and perforation were associated with non-selective embolization of the causative bleeding vessel. Massive hemorrhage requiring an extension of hospital stay and repeated blood transfusions were seen in studies that used streptokinase and heparin.

The study is limited by the lack of studies with high-level evidence. Most studies were observational and likely to overestimate the effects of PMA due to publication bias. Only 10 studies (38.5%) reported the limitations of their study/its design. Significant selection bias may exist, and the patient cohorts were mixed with varying underlying diseases and no concurrent control group. Intra-study variability was also high due to most studies not having a standardized protocol being followed at a single institution and ultimate decision-making being left up to the interventionalist.

## Conclusion

PMA is an important diagnostic and therapeutic tool for identifying and treating the source of obscure GIB when other diagnostic tests have failed. While complications associated with the procedure are generally rare, further studies with high-level evidence and standardized protocols are needed to establish the safety profile of PMA for GIB.

### Supplementary Information

**Additional file 1: Table S1.** Quality assessment. **Table S2.** Treatment details and outcomes- heparin provocation. **Table S3.** Treatment details for studies that used thrombolytics/other agents. **Table S4.** Drug dose details.

## Data Availability

The dataset(s) supporting the conclusions of this article is(are) included within the article (and its additional file(s)).

## Abbreviations

GDA: Gastroduodenal artery
IV: Intravenous
IA: Intra-arterial
SMA: Superior mesenteric artery
IMA: Inferior mesenteric artery
GIB: Gastrointestinal bleeding
IU: International units
PTT: Prothrombin time
tPA: Tissue plasminogen activator
RBC: Red blood cell
Min: Minute
EGD: Esophagogastroduodenoscopy
VCE: Video capsule endoscopy
MR: Magnetic resonance
CT: Computed tomography
NR: Not reported
