# Supplementary material for: Provocative mesenteric angiography for occult gastrointestinal bleeding: a systematic review
Source: CVIR Endovasc. 2023 Aug 17;6:42. doi: 10.1186/s42155-023-00386-7 (PMC10435437; doi:10.1186/s42155-023-00386-7)
Supplement: Supplementary file 1 — Additional file 1: Table S1. Quality assessment. Table S2. Treatment details and outcomes- heparin provocation. Table S3. Treatment details for studies that used thrombolytics/other agents. Table S4. Drug dose details. [file 42155_2023_386_MOESM1_ESM.docx]

**Supplementary information**

**Search terms**

*Boolean operator (AND) was employed in the final search with the following search term combinations.

- "Angiography/methods"[MAJR] OR "Angiography"[MAJR] OR "Angiography, Digital Subtraction/methods"[Mesh] OR "Embolization, Therapeutic"[MAJR] or "Tomography, X-Ray Computed"[mesh] OR “MESENTERIC ANGIO*”
- "Gastrointestinal Hemorrhage/ETIOLOGY"[MAJR] OR "Gastrointestinal Hemorrhage/diagnosis"[MAJR] OR "Gastrointestinal Hemorrhage/diagnostic imaging"[MAJR] OR "Lower Gastrointestinal Tract/diagnostic imaging"[MAJR] OR "Mesenteric Arteries/diagnostic imaging"[MAJR] OR "Mesenteric Artery, Inferior/diagnostic imaging"[MAJR] OR "Mesenteric Artery, Superior/diagnostic imaging"[MAJR] OR "Mesentery/diagnostic imaging"[MAJR] OR "Celiac Artery/diagnostic imaging"[MAJR]
- "Gastrointestinal Hemorrhage/surgery"[MAJR] OR "Gastrointestinal Hemorrhage/therapy"[MeSH] OR "Lower Gastrointestinal Tract/therapy"[MeSH] OR "Lower Gastrointestinal Tract/blood supply"[Mesh] OR "Lower Gastrointestinal Tract/pathology"[Mesh] OR "Mesenteric Arteries/therapy"[MeSH] OR "Mesenteric Artery, Inferior/therapy"[MeSH] OR "Mesenteric Artery, Superior/therapy"[MeSH] OR "Mesentery/therapy"[MeSH] OR "Celiac Artery/therapy"[MeSH] OR "Splanchnic Circulation"[MeSH] OR "VISCERA/BLOOD SUPPLY"[MESH] OR "Colon/pathology"[MESH]
- “Contrast Media"[nm] OR "Heparin"[nm] OR "Tissue Plasminogen Activator"[nm] OR "Vasodilator Agents"[NM] OR "RETEPLASE"[NM] OR "NITROGLYCERIN"[NM] OR "Streptokinase"[Mesh] OR "Tissue Plasminogen Activator"[Mesh] OR "Nitroglycerin"[Mesh] OR "Urokinase-Type Plasminogen Activator"[Mesh] OR "Tolazoline"[Mesh] OR "Vasodilator Agents" [Pharmacological Action] OR "INFUSIONS, INTRA-ARTERIAL"[MESH] OR "RECOMBINANT PROTEINS”[MESH] OR “FIBRINOLYTIC AGENTS”[MESH]

**Table S1: Quality assessment**

|  | A1 | A2 | A3 | A4 | A5a | A5b | A6a | A6b | B9 | C10 | C11 | OLOE |
| --- | --- | --- | --- | --- | --- | --- | --- | --- | --- | --- | --- | --- |
| Bloomfeld *et al.*^25^ | Yes | Yes | Yes | Yes | Can’t tell | Can’t tell | Yes | Yes | Yes | Yes | Yes | 2b |
| Brunnler *et al.*^26^ | Yes | Yes | No | Yes | Can’t tell | Can’t tell | Yes | No | Yes | Yes | Yes | 2b |
| Cohn *et al.*^27^ | Yes | Yes | No | Yes | Can’t tell | Can’t tell | No | No | Yes | Yes | Yes | 2b |
| Kariya *et al.*^9^ | Yes | Yes | Yes | Yes | Can’t tell | Can’t tell | No | No | Yes | Yes | Yes | 2b |
| Kim *et al.*^12^ | Yes | Yes | Yes | Yes | Can’t tell | Can’t tell | Yes | Yes | Yes | Yes | Yes | 2b |
| Kokoroskos *et al.*^31^ | Yes | Yes | Yes | Yes | Can’t tell | Can’t tell | No | No | Yes | Yes | Yes | 2b |
| Koval *et al.*^32^ | Yes | Yes | Yes | Yes | Can’t tell | Can’t tell | Yes | Yes | Yes | Yes | Yes | 2b |
| Lee *et al.*^33^ | Yes | Yes | No | Yes | Can’t tell | Can’t tell | Yes | Yes | Yes | Yes | Yes | 2b |
| Malden *et al.*^34^ | Yes | Yes | Yes | Yes | Can’t tell | Can’t tell | Yes | Yes | Yes | Yes | Yes | 2b |
| Mernagh *et al.*^36^ | Yes | No | Yes | Yes | Can’t tell | Can’t tell | No | No | Yes | Yes | Yes | 2b |
| Nozawa *et al.*^38^ | Yes | Yes | No | Yes | Can’t tell | Can’t tell | No | No | Yes | Yes | Yes | 2b |
| Ryan *et al.* | Yes | Yes | Yes | Yes | Can’t tell | Can’t tell | Yes | Yes | Yes | Yes | Yes | 2b |
| Thiry G *et al.* | Yes | Yes | Yes | Yes | Can’t tell | Can’t tell | Yes | Yes | Yes | Yes | Yes | 2b |
| Widlus *et al.* | Yes | Yes | Yes | Yes | Can’t tell | Can’t tell | Yes | Yes | Yes | Yes | Yes | 2b |
| Zurkiya *et al.*^40^ | Yes | Yes | Yes | Yes | Can’t tell | Can’t tell | No | No | Yes | Yes | Yes | 2b |
| A1, Did the study address a clearly focused issue?; A2, Was the cohort recruited in an acceptable way?; A3, Was the exposure accurately measured to minimize bias?; A4,Was the outcome accurately measured to minimize bias?; A5a, Have the authors identified all important confounding factors?; A5b, Have they taken account of the confounding factors in the design and/or analysis?; A6a, Was the follow-up of subjects complete enough?; A6b, Was the follow-up of subjects long enough?; B9, Do you believe the results?; C10, Can the results be applied to the local population?; C11, Do the results of this study fit with other available evidence? OLOE, Oxford Level of Evidence. | | | | | | | | | | | | |

**Table S2- Treatment details and outcomes- heparin provocation**

| Author | PMA protocol | Injection artery | Complications | Lesion identified |
| --- | --- | --- | --- | --- |
| Nozawa | Heparin (1000IU IA) | SMA/IMA | - |  |
| Hasaj | 3 angiographies with/without heparin provocation- multiple areas of angiodysplasia involving the colon, with no active extravasation, and a calcified splenic artery aneurysm 4 cm in size with no active bleeding.  eventually ERCP was diagnostic followed by embolization of the aneurysm identified on angiography. |  |  | Hemosuccus pancreaticus - hemorrhage through the pancreatic duct into the duodenum -secondary to a ruptured primary splenic artery aneurysm |
| Brünnler | Heparin-provoked scintigraphy- 6 (46%) patients had a positive result with localization of the bleeding, 3 (23%) patients showed a positive result without localization. |  | 3 out of 25 patients dying in the subpopulation of patients with negative scintigraphy results but with no death correlated with the GI bleeding. |  |
| Mernagh | Papaverine, bolus of 5000IU of heparin IV → IV infusion of heparin solution (20,000IU in 500 mL of 5% dextrose in water), started at 31 mL/h and adjusted to maintain the PTT within therapeutic range (60-85s). Heparinized for 24 h | SMA | Nil | Angiodysplasia of the mid-ascending colon (n=1), a vascular malformation of the cecum (n=1), vascular malformations of the small bowel (n=3), and bleeding from an area of active Crohn's disease in the terminal ileum (n=1) |
| Lee | Heparin challenge | - | Massive hematemesis and blood per ileostomy following heparin challenge, requiring substantial blood product transfusion and endotracheal intubation |  |
| Drezdzon | heparin or vasodilators   - CT angiogram showed extravasation at the transverse colon. Formal angiogram was unable to localize the source of bleeding, despite provocation. | - | NA | Multiple adenomas without dysplasia |

**Table S3- Treatment details for studies that used thrombolytics/other agents**

| Author | PMA protocol | Injection artery | Complications | Lesion identified |
| --- | --- | --- | --- | --- |
| Johnston | Sequential boluses of IV heparin (3000 U) and IA papaverine (15 mg), slow infusion of 15 mg IA tPA- failed.  5mg of tPA into the branch of the ileocolic artery- failed.  IMA- sequential injection of 15mg papaverine→ slow infusion of 15 mg tPA IA- success. | SMA, branch of ileocolic artery, IMA | Nil |  |
| Thiry | Nitroglycerin, anticoagulation via heparin, and/or thrombolysis via tPA (0-50mg) | - | Nil |  |
| Miller | Urokinase- no bleeding site- demonstrated mild to moderate atherosclerotic changes | - | - |  |
| Kokoroskos | IV heparin (5000IU) bolus, nitroglycerine (200μg) IA→ angiography after 5min → 10mg of tPA and 200μg of nitroglycerine IA.  Bleeding is still not located→ repeat 10 mg of tPA and 200μg of nitroglycerine IA → angiography after 10 min. | SMA (n=6), IMA (n=1) | Nil |  |
| Koval | Systemic heparinization (5000-10,000 IU) → protamine sulfate  Negative → Tolazoline → Negative →streptokinase → repeat tolazoline.  -The yield of extravasation with heparin alone (2/10 patients) was greatly improved when tolazoline was subsequently administered to those patients who failed to extravasate (5 or 7 patients) | SMA, Celiac artery | Arterial puncture site hematoma, modest post-procedure hemorrhage (n=2).  Transient mild hypotension with tolazoline, two-unit hemorrhage after streptokinase infusion and suffered recurrent bleeding despite three previous laparotomies, six negative angiograms and 150 units of blood transfusion (n=1). | Small bowel angiodysplasia |
| Kim | IV heparin (range, 2,000–6,000 U; mean, 4,286 U), IA tolazoline (range, 12.5– 30 mg; mean, 20.6 mg) or papaverine (n = 1, 1 mg). tPA (alteplase-total mean amount delivered 10.8 mg; range, 1–30 mg) | SMA (n=29), IMA (n=2), sequential SMA/IMA (n=2), left colic (n = 2) or middle colic artery (n = 1) | Embolization-related complication- ischemia and perforation of a segment of ileum after combined particle and coil embolization in the setting of an occult subcentimeter carcinoid tumor (n=1) | Occult subcentimeter carcinoid tumor |
| Ryan | IV heparin (range, 3,000–10,000 U; mean, 4,875 U), IA tolazoline (range, 25– 100 mg; mean, 28.9 mg), and IA tPA (range, 10–50 mg; mean, 20.3 mg). tPA was administered in increments as a slow infusion over the course of 15 minutes.  - provocative mesenteric angiography with tPA for LGI bleeding led to a diagnosis of the site of bleeding in 37.5% of patients and contributed to a decision concerning treatment in 50% of patients. | SMA, IMA, celiac- Decision made to either proceed with further infusion of tPA into the same vessel or proceed to provocation of the second vessel. | Nil |  |
| Kariya | Urokinase (60,000–120,000 IU). Heparin 3000 IU, nicardipine 2 mg, alprostadil 10 μg, isosorbide 1 mg.  - Clinical success was achieved in 5 (83.3%) of the 6 cases in which technical success was achieved after provocative angiography. Clinical success of transcatheter arterial embolization was defined as the achievement of hemostasis for hematochezia with no further transcatheter arterial embolization or surgical treatment. | - | Nil |  |
| Malden | In vitro RBC labeling with technetium → 30 min of scintigraphy → heparin +thrombolytic agent administered. If no bleeding at 30mins, IV bolus of 10,000IU of heparin → continuous infusion of 500IU for 3 hours. Concurrently, given 250,000IU of urokinase and continuous infusion of urokinase for 3 hours. | - | Nil |  |
| Widlus | 5IU Reteplase were diluted to 20 mL with normal saline and injected IA over a 1-minute period. | SMA, IMA | Nil |  |
| Bloomfeld | IA tolazoline (25mg), IA heparin (1,000 to 10,000IU), urokinase in aliquots of 250,000IU over 15-min intervals (up to 1,000,000IU over 1 h). | Celiac artery, IMA? | Continued bleeding at 30 min post-procedure and was treated with intra-arterial vasopressin, which resulted in cessation of bleeding (n=NR) | Diverticular disease in the right colon |
| Rösch | 1) 50mg tolazoline into SMA over 20 seconds, angiography 30 seconds later  2) 10,000IU of heparin to prolong bleeding episode (hematochezia) → 60mg protamine reversal  3) 500IU of heparin in SMA →15 minutes later- 50mg tolazoline → 50mg protamine reversal. Recurrence (7 months later) - 60,000IU of streptokinase in SMA → 50mg of tolazoline. | SMA, celiac artery | Massive hematemesis with streptokinase (n=1) | Ulcer in distal rectum |
| Meade | Combining PMA and EGD- tissue plasminogen activator and heparin- failed to detect a bleeding point. Provocation VCE with systemic heparin, causing melena. Real-time analysis confirmed the duodenum as the bleeding site. PMA with direct injection of tPA and heparin into the GDA and simultaneous OGD were performed under general anesthetic.   - Only after endoscopic confirmation of bleeding was the blush demonstrated on PMA appreciable deriving from a tiny branch of the GDA. | GDA | Nil |  |
| Wu | 2mg of tPA dissolved in 10ccs of saline- 1mg was injected   - aberrant celio-mesenteric branch extending from the CHA to the splenic flexure | Common hepatic artery (CHA) |  |  |
| Remzi | Heparin (5000 U) systemically, tPA (10 mg) was injected during angiography directly into SMA → methylene blue (1 ml) injected selectively through the bleeding artery as distally as possible with the intent of staining the bowel at the point of hemorrhage.   - the site of bleeding was readily identified by the methylene blue staining of the bowel wall. | SMA | Nil | 2-mm mucosal ulceration lying within a narrow band of chronic inflammation |
| George | 3x provocative angiograms- heparin, urokinase, and vasodilators- All negative.   - Intra-arterial scintigraphy was performed by administering 10 mCi of 99mTc-sulfur colloid- showed significant extravasation in the left lower quadrant | SMA | Nil | Mesenteric varices (secondary to portal hypertension due to previously unrecognized alcoholic cirrhosis) |
| Glickerman | Bolus of 10,000IU of heparin via catheter → 1,000 units per hour. After 16 hours of heparin infusion, melena (minimal) → emergency angiography- negative. Heparin infusion continued for 4 more hours, discontinued due to bleeding access site. Melena recurred → received 10,000IU of heparin → angiography- negative → 1 million units of urokinase (rate of 30,000IU per minute) + 25mg of tolazoline – positive bleed | SMA | Bleeding at access site | Multiple focal ectasias in ileum |
| Shetzline | Heparin (500 IU) IV, Priscoline (25mg) into SMA over 1 minute → clearing the catheter with 5mL normal saline → tPA (10mg) infused into SMA over 10 minutes.   - Bleeding in jejunal artery | SMA | Nil |  |
| Zurkiya | Verapamil (100–200 μg) or nitroglycerin (100–300 μg); heparin, 25–100 mg; and a thrombolytic agent such as tissue plasminogen activator, 10–50 mg. Alternatively, heparin may be systemically administered IV with weight-based dosing.   - to localize a lesion for subsequent surgical resection, often in conjunction with administration of methylene blue |  | Nil |  |
| Cohn | Heparin sodium, urokinase, or vasodilators | Celiac artery, SMA, IMA | Incisional hemorrhage secondary to urokinase and heparin administration (n=1) |  |
| Werner | Norepinephrine- intra-arterial, catheter-delivered: The maximum dose *per* patient during an angiography was 40 µg. The maximal individual dose of NE was 10 µg. | SMA, right colic artery, IMA | Extended dearterialization → mesenteric ischemia → right-sided hemicolectomy |  |

**Table S4- Drug dose details**

| Author | Number of patients | Heparin (IU) | tPA (mg) | Others (IU) |
| --- | --- | --- | --- | --- |
| Mernagh | 12 | 5000 |  |  |
| Johnston | 1 | 3000 |  |  |
| Thiry G | 36 |  | 50 |  |
| Nozawa | 2 | 1000 |  |  |
| Kokoroskos | 23 | 5000 | 10 |  |
| Koval | 10 | 10000 |  |  |
| Kim | 36 | 4286 | 10.8 |  |
| Ryan J | 17 | 4875 | 28.9 |  |
| Kariya | 12 | 3000 |  | 120000 -Urokinase |
| Malden | 10 | 10000 |  | 250,000 -Urokinase |
| Widlus | 9 |  | 5 |  |
| Bloomfeld | 7 | 10000 |  | 1,000,000 -Urokinase |
| Rösch | 3 | 10000 |  | 60000 -Streptokinase |
| Wu | 1 |  | 1 |  |
| Remzi | 1 |  | 10 |  |
| Glickerman | 1 | 40000 |  | 1,000,000 -Urokinase |
| Shetzline | 1 | 500 | 50 |  |
| Zurkiya | 19 |  | 50 |  |
| Werner | 4 | - | - | 40µg - Norepinephrine |
